# Diagnostic accuracy of prospectively gated, 128‐slice or greater CTCA at high heart rates: a systematic review and meta‐analysis

**DOI:** 10.1002/jmrs.525

**Published:** 2021-07-07

**Authors:** Gordon T.W. Mander, Karen Dobeli, Caitlin Steffensen, Zachary Munn

**Affiliations:** ^1^ Toowoomba Hospital Darling Downs Health Queensland Health Toowoomba Queensland Australia; ^2^ Faculty of Health Sciences Joanna Briggs Institute University of Adelaide Adelaide South Australia Australia; ^3^ Royal Brisbane and Women’s Hospital Metro North Hospital and Health Service Queensland Health Herston Queensland Australia; ^4^ Philips Australia and New Zealand Brisbane Queensland Australia

**Keywords:** Cardiac computed tomography, computed tomography, diagnostic test accuracy, meta‐analysis, sensitivity and specificity, sinus tachycardia

## Abstract

**Introduction:**

Prospectively gated 64‐slice CT coronary angiography (CTCA) may be contraindicated for heart rates (HRs) over 65 beats per minute (bpm) due to reduced diagnostic sensitivity. Newer CT scanners typically provide 128 or more slices and superior temporal resolution compared with older models; consequently, diagnostic accuracy for current technology prospectively gated CTCA may be adequate at HRs above 65 bpm. The aim of this systematic review was to investigate the diagnostic accuracy of CTCA using 128‐slice or greater CT technology when compared with conventional coronary angiography for patients with HRs >65 bpm.

**Methods:**

A systematic search of PubMed, CINAHL, EMBASE and Scopus was performed as well as unpublished databases, sources and reference lists. Titles and abstracts were screened by two independent reviewers. Full‐text screening was then performed. Studies that determined diagnostic accuracy of coronary artery stenosis in adult patients with high heart rates utilising prospectively gated 128 detector or greater scanners were included. Studies that were included in the review underwent critical appraisal using the QUADAS‐2 tool.

**Results:**

Ten studies were included in the systematic review, with nine of these included in a diagnostic test accuracy meta‐analysis, including six of which reported data at the patient level. Meta‐analysis indicated very high pooled sensitivity 100% (95% CI 0.99, 1.00); however, pooled specificity was less at 79% (95% CI 0.69, 0.88).

**Conclusions:**

Prospectively gated CT coronary angiography may be justifiable at heart rates above 65 bpm if performed on a 128‐slice or greater CT unit. Caution regarding the implication of a positive result is recommended due to reduced specificity. Further evidence is required before consideration of a new higher heart threshold.

## Graphical abstract

High heart rates create challenges for prospectively‐gated CT coronary angiography. A systematic review and meta‐analysis were undertaken to identify the effect of high heart rate (>65 bpm) on diagnostic accuracy. The review found diagnostic sensitivity remains high at higher heart rates 100% (95%CI 0.99, 1.00), however, specificity was shown to be less reliable at 79% (95%CI 0.69, 0.88). Where standard heart rate control is contraindicated or ineffective, prospectively‐gated CT coronary angiography using 128‐slice or greater CT scanners may be justifiable.

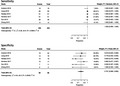

## Introduction

Computed tomography coronary angiography (CTCA) is a well‐established test, primarily due to its excellent negative predictive value in the context of ruling out the presence of coronary artery stenosis.^1^ Guidelines now advocate for the use of CTCA as a frontline test in the assessment of coronary artery disease for low–intermediate‐risk patients ^2^, and there is good evidence to support its use in patients with acute chest pain.^3^

Current recommendations for the performance of CTCA, which are based on data from studies performed using 64‐slice CT technology, include preferential use of a prospectively gated axial scan technique because the radiation dose delivered to the patient is considerably less than that for retrospectively gated helical CTCA. However, prospectively gated CTCA may not be appropriate for patients with high heart rates due to reduced sensitivity resulting from motion artefacts. Thus, current performance guidelines recommend pharmaceutical intervention to maintain heart rate below 65 beats per minute (bpm) during imaging.

Several important technical innovations have been made clinically available over the past decade, which may mitigate the challenges of high heart rate CTCA with 64‐slice CT. These include new iterations of dual‐source technology, as well as other technical improvements such as increased detector coverage and faster gantry rotation speeds. Proprietary software algorithms have also been designed to reduce effects from cardiovascular motion (Snapshot Freeze (GE Healthcare)^4^, ^5^, Adaptive Motion Correction (Canon Medical))^6^. It remains unclear if their implementation has achieved the desired improvement in terms of the diagnostic accuracy of prospectively gated CTCA at high heart rates. There have been no systematic reviews of diagnostic accuracy in CTCA that have focussed on high heart rates since 2013.^7^ Previous recommendations based on superseded technology state that CTCA might be contraindicated for HR over 65 but technology has progressed since then, hence the need for a review of current technology.^8^, ^9^ The aim of this review was to determine the diagnostic accuracy of prospectively gated CTCA performed on 128‐slice or greater multidetector CT (MDCT) for adult patients with high heart rates compared with conventional coronary angiography as a reference standard through a diagnostic test accuracy meta‐analysis.

## Methods

### Inclusion criteria

The review included all study design types describing adult participants (>18 years) with heart rates greater than 65 bpm that did not directly evaluate the effect of known confounding factors such as atrial fibrillation, obesity or intraluminal stents for coronary artery bypass grafts.

Studies that related to the use of MDCT with ≥128 detector rows as the index test and that utilised a prospective‐ECG‐gating mechanism were included. Scans performed using single or multi‐beat reconstructions and any level of temporal padding were included, provided the scan was ostensibly acquired in a prospective ECG (axial) scan mode. Where possible, radiation dose and padding data were collected for comparative purposes. Study cohorts that did not describe traditional or common practices, such as those that looked at novel ECG‐gating techniques, dual‐energy acquisitions or low dose examinations, were excluded. Where a study compared a novel technique to a control arm, only data from the control were included. Where a recognised post‐processing motion correction technique was directly compared with original image data using the same patient group, the former group was included. Only studies that directly compared CTCA with conventional coronary angiography as the reference standard were included.

For the purposes of this review, significant coronary artery disease was defined as a 50% or greater narrowing of a coronary artery segment based on the American Heart Association definition.^10^

### Search strategy

PubMed, CINAHL, EMBASE and Scopus databases were reviewed using a carefully constructed search string. Further to the formal strategy, we also performed searches of ProQuest dissertation database and Google Scholar, and contacted key authors for recommendations. The reference lists of included papers were also screened to identify additional relevant studies that were not discovered during the database searches. Search limits included English language studies only, and dates were limited to 2007 as the technology in question was not clinically available prior to this time. A pragmatic updated search of PubMed conducted on 11/01/2021 did not yield further results.

### Assessment of methodological quality

An assessment of the diagnostic quality of included studies was conducted using the QUADAS‐2 risk of bias and applicability instrument by two of the authors (GTWM and CJS). The assessment was performed independently, and results compared as per the design of the QUADAS‐2 tool.^11^ Where results varied, consensus was achieved through discussion.

### Data collection

Data were extracted from all included papers by the lead reviewer (GTWM). The data extracted included specific details about the tests, populations, study methods and diagnostic accuracy outcomes at patient, vessel and segment levels. The data to be extracted were defined in the review protocol *a priori*.^12^

### Data synthesis

Data were synthesised narratively, and results pooled through proportional paired meta‐analyses.

Meta‐analysis was conducted using a subscription‐based online software package (JBI SUMARI).^13^ Paired forest plots and summary estimates (including 95% confidence intervals) were created for sensitivity and specificity for patient‐level, vessel‐level and segment‐level analyses. Pooled summary estimates were considered superior to receiver operating characteristic (ROC) curves in this setting, as only a single diagnostic threshold (50% coronary artery stenoses) was used.

Summary receiver operating characteristic (SROC) curves were not produced as the review focussed on a single diagnostic threshold, reported 50 per cent narrowing. Summary ROC curves are used in systematic reviews of diagnostic accuracy where diagnostic thresholds vary. Instead, the reviewers felt it was more appropriate to use paired forest plots to determine summary estimates of sensitivity and specificity. Therefore, the summary estimates provided in this review may not be representative if other diagnostic thresholds are used in practice.

Furthermore, positive and negative predictive values were not reported in this review. Whilst the negative predictive value for CTCA is high and is commonly referred to when highlighting the value of the test, care should be taken when interpreting negative predictive values. Predictive values are directly affected by the prevalence of disease in the sample and therefore are not the preferred method of reporting when the reference standard does not necessarily reflect the prevalence expected in clinical practice. For this reason, only sensitivity and specificity values were reported. Sensitivity and specificity are not affected (at least directly) by the prevalence of positive cases in the sample and are therefore considered a more global measure of the true accuracy of CTCA when conventional coronary angiography is used as the reference standard.

Further sensitivity and subgroup analyses were then created using a separate package (RevMan v5.3, Copenhagen, Denmark). Paired forest plots were generated for these analyses; however, pooled summary estimates were not created for sensitivity and specificity as there were insufficient data to perform this.

## Results

### Search results

**Figure 1:**
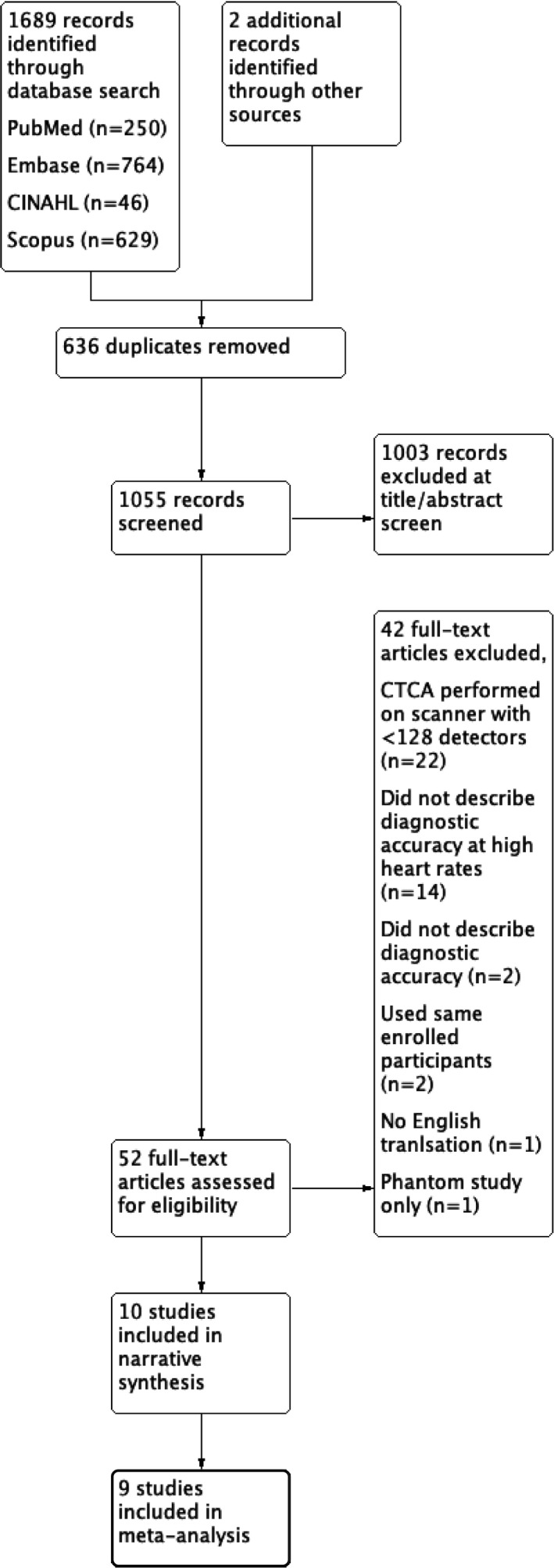
PRISMA flow diagram.

Database searches identified 1689 records (Figure 1). Following title and abstract screening, 53 full‐text articles were assessed for eligibility against the detailed inclusion criteria for the review. An additional two records were identified outside of the database strategy; one through the reference list of included studies and the other through contact with primary study authors. Ultimately, 10 studies were included in a narrative synthesis, with nine of these studies further included in a meta‐analysis.

### Characteristics of included studies

Overall, data from 450 participants were captured in the results of this review. Six of the 10 included studies were performed in China with the other studies performed in each of Australia, Italy, Turkey and the Netherlands. Only studies that evaluated patients with HR >65 bpm were included in the review. However, some studies evaluated high heart rates only, whilst others performed a subgroup analysis for these patients. Heart rates for participants in each included study are provided in Table 1.

**Table 1:** Heart rates for participants included in diagnostic accuracy assessment.

Study (First Author DATE)	All Participants in Study Population (including Low HR)					High Heart Rate Group				
	No. of Participants (*N*)	Heart Rate (mean) (bpm)	Standard Deviation (SD)	HR Range (bpm)		No. of Participants (*n*)	Heart Rate (mean) (bpm)	Standard Deviation (SD)	HR Range (bpm)	
				Min.	Max.				Min.	Max.
Andreini 2018^4^	100	N/A	N/A	N/A	N/A	40	93	±23.6	81	Unclear^†^
Gang 2012^21^	60	73.7	±15.4	51	128	26	86.5	±15.1	73	128
Li 2013^17^	N/A	N/A	N/A	N/A	N/A	61	75	±7.7	65	80
Liang 2019^14^	N/A	N/A	N/A	N/A	N/A	81	83.8	±8.9	75	134
Neefjes 2013^18^	267	65	±12	N/A	N/A	67	75	±12	65	Unclear^†^
Nerlekar 2017^15^	107	N/A	N/A	37	80	52	69*	±8	60	80
Selçuk 2016^19^	102	64	±4	44	102	Unclear^†^	Unclear^†^	Unclear^†^	70	102
Sun 2013^16^	N/A	N/A	N/A	N/A	N/A	47	79	±9	66	100
Wang 2016^22^	100	76.44	±13.36	39	107	60	Unclear^†^	Unclear^†^	75	107
Zhang 2016^20^	43	69.4	±13.6	45	106	16	Unclear^†^	Unclear^†^	70	106

\x90* Result includes participants in study that were excluded from review.

† *‘*Unclear’ recorded if data were studied but not reported. N/A is recorded if not studied.

The review considered studies from all vendors including; three studies based on Aquilion ONE and Aquilion ONE Vision (Canon Medical, Japan) scanners, two studies that utilised Revolution CT (GE Healthcare, Waukesha, Wisconsin, USA) scanners, four studies that described Somatom Definition Flash (Siemens Healthineers, Forchheim, Germany) scanners and one study that assessed a Brilliance iCT (Philips Healthcare, Best, Netherlands) scanner. All studies reported prospective‐ECG acquisition techniques; however, the level of temporal padding employed differed between studies, as did corresponding median dose‐length product (DLP) values for each study.

Table 2 details the scanner characteristics associated with each of the included studies.

**Table 2:** Heart rates for participants included in diagnostic accuracy assessment.

Study	Make	Model	Scan Parameters						
			Padding		Segment	Detector Row	Rotation Time (ms)	Motion Correction	Dose‐Length Product (DLP) (mGy)
			Start	End					
Andreini 2018^4^	GE Healthcare	Revolution CT	40%	80%	Single beat	256 × 0.625	280 ms	SnapShot Freeze	209
Gang 2012^21^	Canon Medical*	Aquilion ONE	30%	90%	Multi‐beat	320 × 0.5	350 ms		779^†^
Li 2013^17^	Canon Medical*	Aquilion ONE	30%	80%	Multi‐beat	320 × 0.5	350 ms		321^†^
Liang 2019^14^	GE Healthcare	Revolution CT	30%	60%	Single beat	256 × 0.625	280 ms	SnapShot Freeze	70
Neefjes 2013^18^	Siemens Healthineers	SOMATOM Definition Flash	55%	Unclear	Single beat	2 × 64 × 0.6	280 ms		Unclear
Nerlekar 2017^15^	Canon Medical*	Aquilion ONE ViSION Edition	30%	80%	Single beat	320 × 0.5	275 ms		193
Selçuk 2016^19^	Siemens Healthineers	SOMATOM Definition Flash	60%	‐	Multi‐beat	2 × 64 × 0.6	280 ms		Unclear
Sun 2013^16^	Siemens Healthineers	SOMATOM Definition Flash	20%	Unclear	Unclear	2 × 64 × 0.6	280 ms		61
Wang 2016^22^	Philips Healthcare	Brilliance iCT	N/A	N/A	N/A	256 × 0.6	N/A		N/A
Zhang 2016^20^	Siemens Healthineers	SOMATOM Definition Flash	20%	Unclear	Single beat	2 × 64 × 0.6	280 ms		Unclear

\x90* (formerly Toshiba Medical).

† Estimate converted to dose‐length product from effective dose using the dose coefficient included in the study method.

All studies reported significant coronary artery disease as >50% stenosis. Only data at this diagnostic cut point were included in the analysis. The reported sensitivity and specificity for each included study at patient, vessel and segment levels are included in Table 3.

**Table 3:** Narrative synthesis reported sensitivity and specificity by each included study.

Study	Patient level		Vessel Level		Segment Level	
	Sens (%) (95% CI)	Spec (%) (95% CI)	Sens (%) (95% CI)	Spec (%) (95% CI)	Sens (%) (95% CI)	Spec (%) (95% CI)
Andreini 2018^4^	100*	81.8 (65.7–97.9)*	N/A	N/A	95.2 (93.6–96.9)	98.9 (98.1–99.7)
Gang 2012^21^	N/A	N/A	N/A	N/A	94.6 (85.13–98.88)	97 (94.38–98.62)
Li 2013^17^	97 (84.7–99.5)	89.3 (72.8, 96.3)	91.1(79.3–96.5)	96.5(93.0–98.3)	95.5 (90.9–97.8)	98.0 (96.7–98.8)
Liang 2019^14^	100^‡^	85.7^‡^	96.6^‡^	96.6^‡^	92.2^‡^	97.8^‡^
Neefjes 2013^18^	100 (93.0–100 95)	63 (35–85)	99 (96–100)	84 (78–89)	93 (88–98)	93 (91–95)
Nerlekar 2017^15^	100 (90–100)	88 (64–99)	98 (91–100)	94 (89–97)	84 (76–90)	96 (94–97)
Selçuk 2016^19^	87.8^‡^	88^‡^	81.4^‡^	95^‡^	87.8^‡^	99.2^‡^
Sun 2013^16^	100 (88.0–100)	63.6 (31.6–87.6 )	90.0 (81.4–95.0)	95.2 (91.9– 97.2)	92.6 (86.1–96.4)	97.0 (95.1–98.2)
Wang 2016^22^						
(HR 70–90 bpm)	N/A	N/A	N/A	N/A	96.00^‡^	93.70^‡^
(HR>90 bpm)	N/A	N/A	N/A	N/A	97.60^‡^	92.20^‡^
Zhang 2016^20^	100 (73.2–100)	100 (19.8–100)	96.4 (80.0–99.8)	91.7 (76.4–97.8)	88.6 (74.6–95.7)	90.8 (84.8–94.7)

\x90* Result based on evaluable segments only (non‐diagnostic segments excluded).

‡ 95% confidence interval not reported.

### Assessment of methodological quality

**Figure 2:**
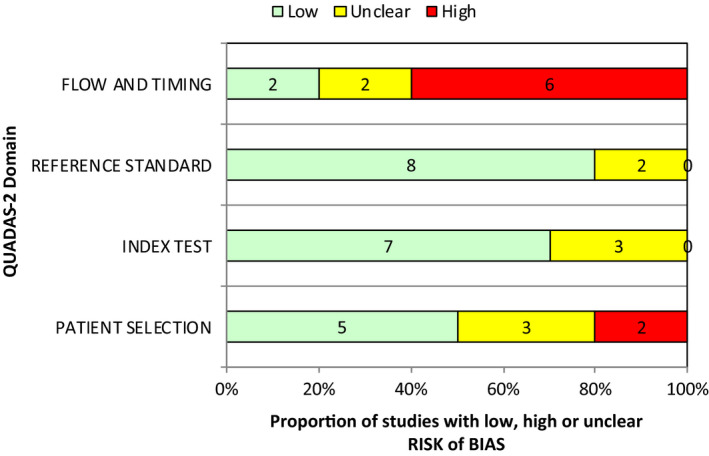
QUADAS‐2 Risk of Bias assessment.

Overall, methodological quality was rated as high. Issues associated with the flow and timing of the study design and patient selection were noted. Proportional results for the methodological assessment are shown in Figure 2. This was due to the concern the reviewers held regarding spectrum bias and partial verification bias in the included studies. In the case of risk of spectrum bias, this was due to a convenience or non‐random sample of patients receiving the reference standard. Risk of partial verification bias was considered high where a study excluded a patient from the analysis.

### Findings of review

#### Narrative synthesis

All included studies were cross‐sectional designs. The majority of studies performed the ICA prior to CTCA as this ensured that only patients receiving both tests were being independently assessed for diagnostic accuracy.

Four primary studies directly analysed the diagnostic accuracy of patients undergoing CTCA with high HRs.^4^, ^14^, ^15^, ^16^

Four studies that reported patient‐level diagnostic accuracy data were included in the review.^17^, ^18^, ^19^, ^20^ Whilst the primary aim of these studies was not to directly study high HRs, they provided subgroup data directly pertinent to the review question.

The remaining two studies included in the review reported diagnostic accuracy at segment level only. Gang and colleagues enrolled consecutive patients with high‐risk coronary artery disease to receive clinically mandated CTCA and then ICA.^21^ A subgroup of enrolled patients with a heart rate greater than 70 bpm was reported, and these data were collected for the review. Whilst the authors reported patient‐level accuracy results overall, this was not reported for the high heart rate subgroup.

Wang and colleagues reported segment‐level data in their assessment of patients with various heart rates.^22^ Three subgroups of patients were compared as follows: patients with low heart rates less than 75 bpm, patients with heart rates between 75 and 90 bpm and those with heart rates greater than 90 bpm. The authors reported sensitivity and specificity in the latter two groups as 96.0% and 93.70%, and 97.60% and 92.20%, respectively. Confidence intervals were not provided for these results.

Overall, the literature provides evidence of high sensitivity results for patients with high heart rates with point estimates of sensitivity varying between 87.8% and 100%. Specificity is more variable, with included studies reporting estimates between 63% and 100%.

Diagnostic accuracy at the vessel‐ and segment‐level accuracies is summarised in Table 3.

#### Meta‐analysis

**Figure 3:**
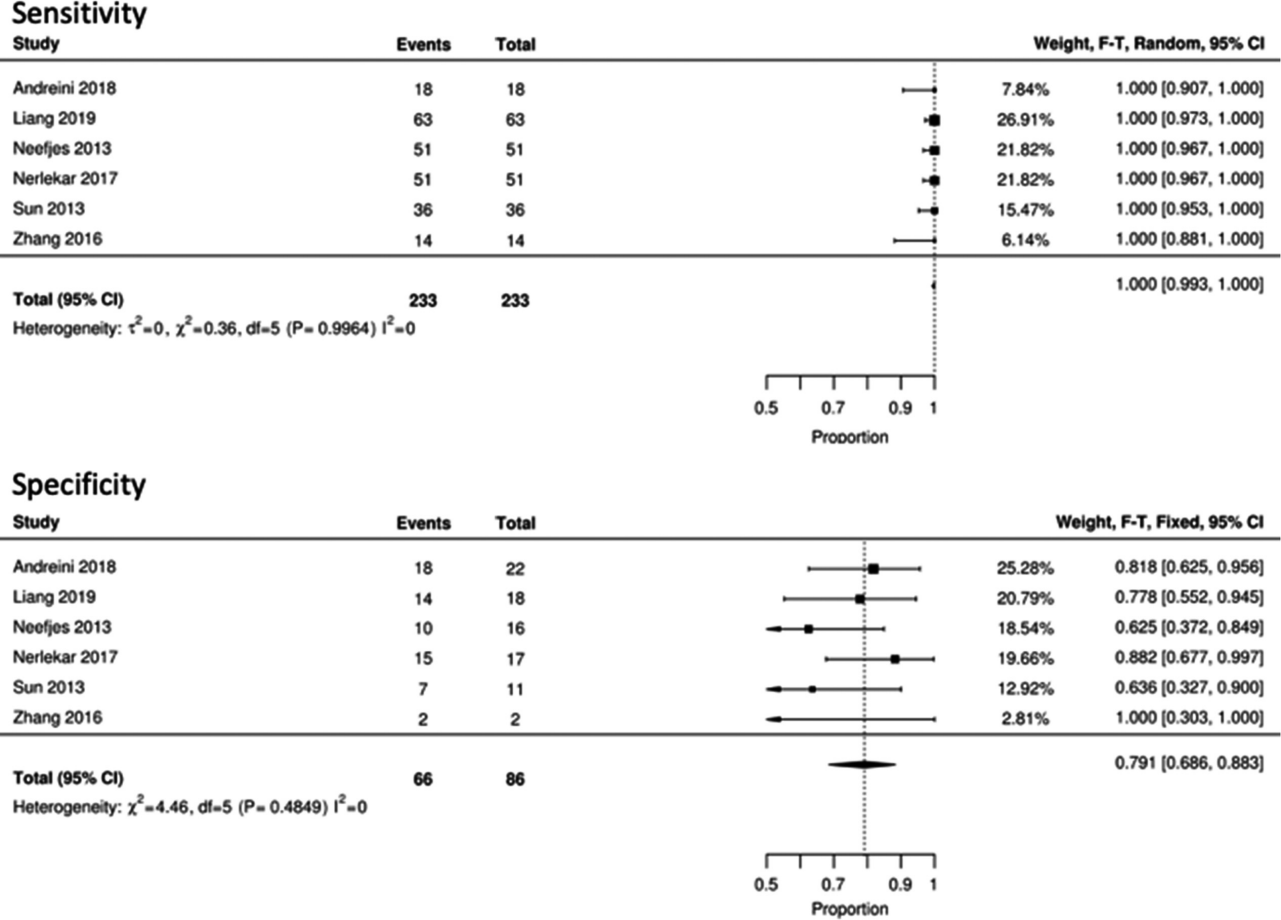
Diagnostic accuracy meta‐analysis for patients with high heart rates.

Nine of the 10 included studies provided sufficient data to perform meta‐analysis. Insufficient data were provided by one study, and it was therefore excluded from the analysis.^19^ Of the remaining studies, six contained patient‐level accuracy information and were included, as shown in Figure 3.

Paired proportional meta‐analysis was performed for all included data at the patient (*n* = 450), vessel (*n* = 1229) and segment levels (*n* = 8144). Overall sensitivity was 100% (95% CI: 0.99, 1.00) and specificity 79% (95% CI 0.69, 0.88). For the vessel‐level analysis, sensitivity was 96% (95% CI: 0.93, 0.97) and specificity 93% (95% CI: 0.90, 0.96). For the segment‐level analysis, sensitivity was 91% (95% CI: 0.88, 0.93) and specificity 96% (95% CI: 0.95, 0.98). The paired meta‐analyses are included as Figure 3.

#### Sensitivity analysis

Most of the included papers reported non‐diagnostic segments as positive to reflect clinical implications of an equivocal result. However, three studies excluded these data. Therefore, a sensitivity analysis was performed to identify the effect positive threshold has on the summary estimates of sensitivity and specificity at the segment level.

At the segment level, an analysis including only studies that treated indeterminate segments as positive produced sensitivity of 90% (95% CI: 86%, 93%) and specificity of 96% (95% CI: 94%, 98%). Compared with the all‐inclusive sensitivity and specificity values of 91% and 96%, respectively, this adjusted result does not indicate a substantial variation in the summary estimates provided. Furthermore, where data were re‐analysed incorporating only studies that had excluded indeterminate segments from the analysis, sensitivity was 93% (95% CI: 90%, 95%), and specificity was 98% (95% CI: 97%, 99%). Again, no substantial difference exists.

#### Subgroup analysis

**Figure 4:**
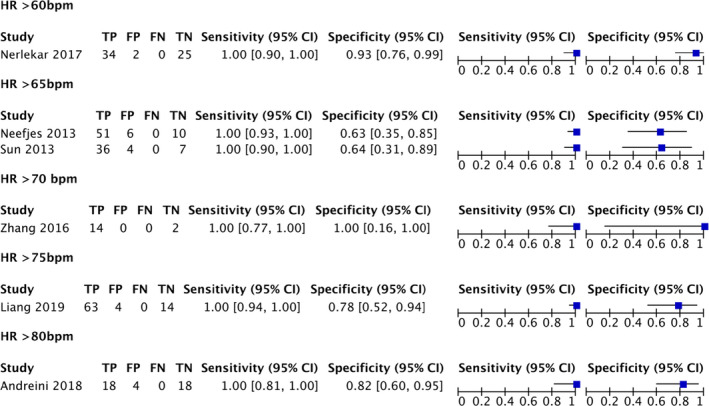
Paired forest plot subgroup analysis of minimum heart rate thresholds.

A subgroup analysis was originally planned to assess for the effect‐specific heart rate had on test accuracy. However, there were insufficient individual data points to perform this analysis. Instead, a subgroup analysis based on the minimum heart rate reported for each of the primary studies was included (Figure 4).

**Figure 5:**
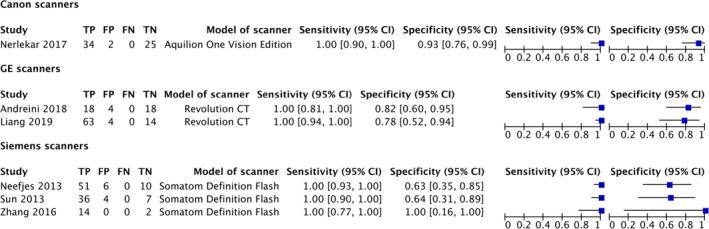
Subgroup analysis of scanner manufacturer.

Scanner make and model subgroup analysis performed and provided as Figure 5. Summary estimates were not reported for either of the subgroup analyses, as there was insufficient power to provide a significant result. No studies provided sufficient data for Philips make scanners at the patient level.

## Discussion

### Interpretation of results

Several previous systematic reviews have assessed diagnostic accuracy of CTCA;^23^, ^24^, ^25^, ^26^, ^27^ however, this is the first to directly assess the effect high heart rate has on diagnostic performance when utilising latest generation technology.

A 2013 systematic review investigated the diagnostic accuracy of CTCA across several ‘difficult‐to‐image’ patient groups.^7^ The review considered several factors believed to affect diagnostic accuracy: obesity, high calcium score, arrhythmias, high heart rate and previous stent or coronary artery bypass graft. For patients with high heart rates (>65 bpm), the review described accuracy with 97.7% sensitivity and 86.3% specificity. These summary estimates fall within the 95% CI of our review. Despite our review describing a more conservative estimate for diagnostic specificity, the higher pooled sensitivity reported in this review (100%) suggests more recent developments in scan technology have further improved sensitivity at high heart rates.

Although the subgroup analysis did not have statistical power to provide a definitive conclusion on the value of particular scan technologies on the diagnostic accuracy at high heart rates, the results reported by each of the individual studies were similar, indicating that each individual scan technology is valuable to ensure accuracy at higher heart rates. However, further study is needed to provide a definitive result for each group.

Whilst the included padding and subsequent dose‐length product (DLP) values for included studies were summarised, they were not a primary outcome of the review. It is assumed that the amount of padding, and therefore dose, incorporated will increase the likelihood of a more accurate outcome; however, the relationship is unlikely to be linear, due to several other confounding factors. The amount of padding used in the included varies between studies and is likely to be higher than that commonly encountered in clinical practice.

It is recognised that the reported sensitivity and specificity are different for patient‐, artery‐ and artery segment‐level analyses. This is explainable by the nature of how each group are calculated. That is, only one of three arteries needs to have a significant stenosis present to report a positive finding for a patient. In comparison, at the vessel–segment level a positive can only be recorded where there is disease in that segment. This effect increases the sensitivity (true positive rate) and decreases the specificity (true negative rate) of patient‐level analyses relative to the vessel–segment analyses, and the phenomena have been reported previously.^23^

### Limitations of the review

A limitation in this review was a paucity of individual data points that could be extracted from the primary studies to allow for a more comprehensive assessment of the effect of heart rates on diagnostic accuracy. Considerable effort was made to contact each study author to enquire about how certain variables, such as the discrete heart rates and padding used, differed between study participants. However, without this information, the scope of the review was limited to determining the current state of play in accurate diagnosis of CTCA, rather than exploring in further detail the strength of particular factors, such as the effect of discrete heart rates and vendor‐specific technology on test accuracy.

As only 6 studies were included in the patient‐level meta‐analysis, comment is made that the results may be limited. However, due to the similarity in the individual study results and relatively small confidence intervals in the summary estimates, it is surmised there is sufficient statistical power to produce a meaningful and accurate result. Furthermore, a lack of primary data is a limitation of the level of exploration of this field of study, rather than a limitation of the review methodology itself. We therefore recommend further research to explore the effect of high heart rates on the accuracy of diagnosis.

The temporal padding differed significantly between and within studies. Whilst this has the potential to increase heterogeneity of the review, the reviewers felt variation in the amount of padding used was primarily controllable by the staff performing the scan. Consequently, it is expected to vary across patients. Likewise, the radiation dose, measured as the dose‐length product, was also considered to have too many resulting factors to consider this a significant source of clinical heterogeneity between the studies.

Studies based on retrospectively gated helical CTCA were excluded from this systematic review. Although helical CTCA using 128‐slice or greater CT may show similar, or even better diagnostic accuracy compared with prospectively gated axial CTCA at high heart rates, the decision to exclude helical CTCA from this study was made to provide a more focussed discussion of diagnostic accuracy between older and newer CT technologies.

Whilst studies that exclusively studied difficult‐to‐image patients were excluded, it was not necessarily clear that, for example, coronary calcium blooming or heart rate variability was not still a cause for misdiagnosis in the data. This is a concern in all data sets where CTCA accuracy is being assessed; consequently, it can be assumed it would not have a significant impact on the results presented here.

There was a significant degree of complexity in selecting included participants for this review. Although several of the included studies directly identified the diagnostic accuracy of high heart rates within their studies, studies were also included where only a small subgroup of patients who underwent the test had high heart rates. Additionally, because the review excluded scans that were performed using retrospective ECG gating, only data where it was clear prospective‐gating had been used (with or without padding) were included.

The inclusion of the study by Nerlekar and Colleagues^15^ constitutes a deviation from the review protocol, as the group reported on high heart rate where the lower inclusion limit was 60 bpm (compared with 65 bpm, stated for this review). The decision was made to include this study as the investigation still related to the effect of high heart rates on diagnostic accuracy. Furthermore, there was no discernible heterogeneity evident between results of this study and others in the review. No studies reporting high heart rates were excluded based on this criterion.

Finally, there was some concern regarding the populations of three studies initially included in the review by Liang and Colleagues.^5^, ^14^, ^28^ The authors reported different enrolment dates for the participants in each of the three studies. However, following communication with the authors it was discovered that all three studies used some or all of the same participants. As the type of reconstruction technology was different in the three studies, only the most recent 2019 study was incorporated in the meta‐analysis as this was thought to provide the most useful data on current technology.

### Implications for practice

Scanning at high heart rates using retrospectively gated axial acquisition on 128‐slice or greater MDCT is appropriate and the diagnostic value maybe non‐inferior to imaging at low heart rates. However, consideration must be given to a likely decrease in resultant specificity for high heart rate patients. Therefore, scanning at high heart rates should still only be considered where appropriate heart rate lowering medications is inappropriate and where the scan is used to rule out rather than evaluate clinically significant coronary artery stenoses.

## Conclusion

This systematic review describes high level accuracy for patients undergoing CTCA with high heart rates, when comparing against ICA as the reference standard. Diagnostic test accuracy paired meta‐analysis produced sensitivity and specificity summary estimates of 99% (95% CI: 0.99, 1.00) and 79% (95% CI: 0.68, 0.88) for patient level, 96% (95% CI: 0.93, 0.97) and 93% (95% CI: 0.90, 0.96) for vessel level, and 91% (95% CI: 0.88, 0.93) and 96% (95% CI: 0.95, 0.98) for segment level.

Further study is required to better understand the effect that individual vendor‐specific technologies have on diagnostic performance, particularly for historically difficult‐to‐image groups such as patient with high HR.

## Conflicts of Interest

CJS is a current employee of Philips Australia and New Zealand. No further conflicts of interest are identified.
